# Metabolic stress in cancer cells induces immune escape through a PI3K-dependent blockade of IFNγ receptor signaling

**DOI:** 10.1186/s40425-019-0627-8

**Published:** 2019-06-13

**Authors:** Koen A. Marijt, Marjolein Sluijter, Laura Blijleven, Sofie H. Tolmeijer, Ferenc A. Scheeren, Sjoerd H. van der Burg, Thorbald van Hall

**Affiliations:** Department of Medical Oncology, Oncode Institute, C7-P, Leiden University Medical Center, Albinusdreef 2, Leiden, 2333 ZA the Netherlands

**Keywords:** Cancer metabolism, Tumor microenvironment, Immune-escape

## Abstract

**Background:**

T-cell mediated immunotherapy brought clinical success for many cancer patients. Nonetheless, downregulation of MHC class I antigen presentation, frequently occurring in solid cancers, limits the efficacy of these therapies. Unraveling the mechanisms underlying this type of immune escape is therefore of great importance. We here investigated the immunological effects of metabolic stress in cancer cells as a result of nutrient deprivation.

**Methods:**

TC1 and B16F10 tumor cell lines were cultured under oxygen- and glucose-deprivation conditions that mimicked the tumor microenvironment of solid tumors. Presentation of peptide antigens by MHC class I molecules was measured by flow cytometry and via activation of tumor-specific CD8 T cell clones. The proficiency of the IFNy-STAT1 pathway was investigated by Western blots on phosphorylated proteins, transfection of constitutive active STAT1 constructs and qPCR of downstream targets. Kinase inhibitors for PI3K were used to examine its role in IFNy receptor signal transduction.

**Results:**

Combination of oxygen- and glucose-deprivation resulted in decreased presentation of MHC class I antigens on cancer cells, even in the presence of the stimulatory cytokine IFNy. This unresponsiveness to IFNy was the result of failure to phosphorylate the signal transducer STAT1. Forced expression of constitutive active STAT1 fully rescued the MHC class I presentation. Furthermore, oxygen- and glucose-deprivation increased PI3K activity in tumor cells. Pharmacological inhibition of this pathway not only restored signal transduction through IFNy-STAT1 but also improved MHC class I presentation. Importantly, PI3K inhibitors also rendered tumor cells sensitive for recognition by CD8 T cells in culture conditions of metabolic stress.

**Conclusions:**

These data revealed a strong impact of metabolic stress on the presentation of tumor antigens by MHC class I and suggest that this type of tumor escape takes place at hypoxic areas even during times of active T cell immunity and IFNy release.

**Electronic supplementary material:**

The online version of this article (10.1186/s40425-019-0627-8) contains supplementary material, which is available to authorized users.

## Background

Recent advances in immunotherapy have led to great clinical successes in multiple cancer types, especially advanced melanoma and non-small cell lung carcinoma. T cell based immunotherapy, like checkpoint blockade therapy, adoptive T cell transfer, and DC vaccination all rely on the surface presentation of tumor-associated or tumor-specific (neo-)antigens on cancer cells by major histocompatibility complex class I (MHC-I) molecules [1, 2]. Type II interferon-y (IFNy) is a major regulator of MHC-I gene expression and, in addition, also upregulates the expression of processing associated genes, like immunoproteasome subunits and the peptide transporter TAP [3]. Therefore, proficient IFNy signaling in the local tumor microenvironment (TME) is of utmost importance for immune-surveillance by the adaptive immune system and scrutiny of intracellular tumor antigens. Many cancer types downregulate MHC-I surface display as primary or acquired immunological resistance through molecular mechanisms affecting IFNy signaling, including loss-of-function mutations in the Janus kinase (JAK) signal transducers, mutations in the IFNy receptor, and alterations in APLNR and PTPN2 genes that control IFNy sensing [4–8]. However, the impact of tumor microenvironmental metabolic cues on IFNy receptor signaling and immune recognition of cancer cells is a largely unexplored area of research.

Due to unregulated tumor growth and uncontrolled angiogenesis, human solid cancers often contain a dysfunctional vascular system, restricting the availability of essential nutrients like oxygen and glucose, triggering metabolic stress in the tumor cells [9, 10]. Oxygen levels in solid cancers often drop to levels between 0.3 and 4.2% (2–32 mmHg) with most below 2% [11–15]. When cells experience hypoxic stress, HIF1a degradation is prevented via prolyl hydroxylases (PHDs) inhibition, leading to induced expression of transcription factors responsible for modulating energy metabolism by activation of the glycolysis and glutaminolysis pathway, including upregulation of the glucose transporter GLUT-1 [16] as well as the glycolytic enzymes Hexokinase-1 (HK-1), Hexokinase-2 (HK-2), and Phosphoglycerate kinase-1 (PKG1) [17–20]. Enhanced glycolysis leads to increased consumption of glucose by cancer cells, draining glucose from the interstitial fluid, resulting in very limited glucose concentrations in the TME. Glucose concentrations in the interstitial fluid of two mouse melanomas reported in situ glucose levels of approximately 0.6 mM compared to 9 mM in the spleen and blood [21], similar to observations reported for other tumor types [22, 23]. Importantly, these microenvironmental nutrient limitations correlate with poor patient survival [24]. Several studies investigated the effects of nutrient limiting conditions on immune cells infiltrating the TME [21, 25]. However, the effects of nutrient deprivation on cancer immunogenicity remain poorly understood.

In this study, we examined the impact of limited oxygen- and glucose- levels as found in the TME, on MHC-I presentation by cancer cells. Under these circumstances cancer cells lose their sensitivity to IFNy by disrupting the IFNy-STAT1 signaling pathway and consequently antigen presentation via MHC-I molecules. Mechanistically, this was related to the activation of the PI3K pathway and pharmacological inhibition of PI3K, using small molecule inhibitors, restored functional MHC-I antigen presentation under oxygen- and glucose-deprived conditions. Our findings describe a novel immune escape mechanism how cancer cells can acquire resistance for immunotherapy.

## Material and methods

### Cell culture

Tumor cells were seeded in 6-well plates and cultured overnight until fully attached (t_0_). Next, tumor cells were cultured under normal (21% oxygen, 25 mM glucose), glucose deprivation (GD, 21% oxygen, 0.5 mM glucose), oxygen deprivation (OD, 1% oxygen, 25 mM glucose), or oxygen and glucose deprivation (OGD, 1% oxygen, 0.5 mM glucose) conditions with, our without IFNy (20 U/mL) for 24 h. Tumor cell medium (TCM) contained IMDM media (Invitrogen) supplemented with 100μg/mL streptomycin, 100 U/mL penicillin, 2 mM L-glutamine (Invitrogen) and 10% FCS (Gibco). Tumor cells were maintained in humidified air at 37 °C and with 5% CO_2_. Pharmacological inhibition of PI3K was done by adding LY294002 (Seleckchem) or wortmannin (Seleckchem) at timepoint t_0_ for 24 h.

T cell medium contained IMDM media (Invitrogen) and 10% FCS (Greiner), 0.2% β2ME, 100μg/mL streptomycin, 100 U/mL penicillin, 2 mM L-glutamine (Invitrogen), and 10 IU/mL recombinant human IL-2 (Novartis). B16F10 specific CD8 T cells (CTL clone LP9 [26]) were stimulated once a week with irradiated B16F10-B7 tumor cells and splenocytes from C57BL/6 mice. TC1 specific CD8 T cells (CTL clone 9.5c3 [27]) were stimulated once a week with irradiated TC1-B7 tumor cells and splenocytes from C57BL/6 mice.

### Functional T cells assays

B16F10 specific (CTL clone LP9, K^b^-binding TRP2_180–188_) [26] or TC1 specific (CTL clone 9.5C3, D^b^-binding HPV16/E7_49–57_) [27] T cells were co-cultured overnight under normal culture conditions, with either B16F10 or TC1 tumor cells collected from normal, OD, GD, or OGD conditions of which MHC-I antigen presentation was fixated using 1μg/mL GolgiPlug (BD Biosciences). T cells were incubated with 1μg/mL TRP2 peptide and used as positive control. After 24 h incubation T cells were fixed, permeabilized, and stained for IFNγ and analyzed by flow cytometry.

### RNA isolation & cDNA synthesis

Tumor cells were washed with cold PBS followed by total RNA isolation using the RNeasy Plus Mini Kit (Qiagen). 1000 ng of RNA was used for cDNA synthesis using a High Capacity RNA-to-cDNA kit (Applied Biosystems).

### qPCR gene expression analysis

qPCRs were performed using the CFX96TM Real Time System (Biorad) with SYBR® Green Supermix (Biorad). The cycling conditions were set at 95 °C for 3 min, followed by 40 cycles of 15 s at 95 °C, 30 s at 60 °C and 30 s at 72 °C. Measurements were performed in triplicates. Ct-values were normalized to the expression of the housekeeping gene UBC. Primer sequence overview can be found in Additional file 2: Table S1.

### Flow cytometry

Tumor cells were stained with H-2D^b^ (28-14-8), or H-2K^b^ (AF6–88.5) antibodies for 30 min at 4 °C. T cell activation was quantified by measuring intracellular IFNγ (XMF1.2, Biolegend) production using an ICS kit (BioLegend) according to manufactures protocol. Cells were acquired on a Fortessa flowcytometer (BD Biosciences) and analyzed using FlowJo software (Tree Star).

### Microscopy

An Olympus DX51 light microscope and Olympus cellSens software was used to capture images of tumor cells cultured under normal, OD, GD, or OGD conditions.

### Western blot

Cellular protein extracts were isolated by directly adding Laemmli sample buffer containing 5% beta mercaptoethanol to the cells. Protein concentrations were measured using Bradford protein assay. Equal amounts of protein extracts (50μg) were separated by Mini Protean precast gels TGX (Biorad). Afterwards, protein extracts were transferred to nitrocellulose membrane using a Trans-Blot® TurboTM transfer Pack (biorad) and Biorad semi-dry Transfer System. Membranes were blocked for 1 h in 5 mL 5% BSA/0.1% Tween/TBS and probed overnight with primary antibody for pSTAT1 Y701 (Cell Signaling #9167), pSTAT1 S727 (Cell Signaling #9177), Total-STAT1 (Cell Signaling #9172), IRF-1 (Cell Signaling #14028), Phospho-AKT (Cell Signaling #9271) or beta-actin (Cell Signaling #3700). membranes were washed 3 times with 5 mL 0.1% Tween/TBS and incubated with anti-rabbit IgG or anti-mouse IgG with HPR-linked antibody (Cell signaling #7074, #7075) for 1 h. Membranes were developed using 1X SignalFireTM ECL Reagent (Cell signaling) and analyzed with a compatible imager. Western-blots were quantified by measuring protein band intensity using imageJ software. Samples were normalized with beta-actin band intensity.

### Statistical analysis

All data are presented as means and SD. Statistical analysis was done using a paired Student’s t test (two-tailed) with welch correction to determine the statistical significance of the differences. A minimum of three replicates were used in all experiments. Differences were considered statistically significant at *p* < 0.05. (* *p* < 0.05, ** *p* < 0.01, *** *p* < 0.001).

## Results

### Oxygen- and glucose-deprived conditions downmodulate MHC-I surface expression on tumor cells resulting in immune escape from CD8 T cells

**Fig. 1:**
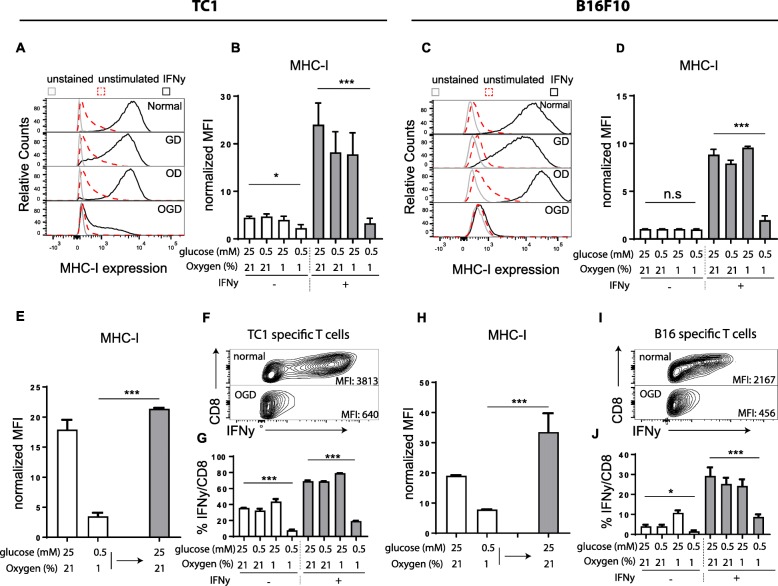
OGD culture conditions impairs IFNy response and leads to abrogated tumor recognition by CD8 T cells. **a** and **c** Histogram plots of MHC-I expression of TC1 (**a**) and B16F10 (**c**) when cultured under different nutrient limiting conditions with our without stimulation of IFNy for 24 h. **b** and **d** Quantification of MHC-I expression of TC1 (**b**) and B16F10 (**d**) tumor cells of three independently performed experiments. Data is shown as normalized Mean Fluorescence Intensity (MFI) by dividing the MFI of the stained sample by the MFI of negative control. **e** and **h** B16F10 (**e**) or TC1 (**h**) tumor cells were cultured under normal or OGD conditions for 24 h with IFNy (white bars). OGD cultured tumor cells were cultured an additional 24 h under nutrient repleted conditions (gray bars). MHC-I surface expression of tumor cells was measured by flow cytometry. Quantitative data of three independent experiments is shown as normalized MFI. **f** and **i** Density plots of IFNy production of TC1 (**f**) or B16F10 (**i**) specific CD8 T cells upon tumor recognition measured by flow cytometry. MFI, mean fluorescence intensity. **g** and **j** Quantified data of (**f** and **i**). Data is shown as mean + − SD. Unpaired t test was used to calculate significance; n.s., not significant *, *p* < 0.05, **, *p* < 0.01, ***, *p* < 0.001

Here we set out to investigate the role of metabolic stress on MHC-I expression levels on tumor cells. Therefore, we cultured the HPV16 E6 and E7 transformed TC1 cancer line and the B16F10 melanoma line under nutrient proficient (normal), oxygen deprived (OD) (1% oxygen), glucose deprived (GD) (0.5 mM), or the combination of oxygen- and glucose-deprived (OGD) conditions, simulating the physiological levels measured in the microenvironment of solid cancers [11, 21–23]. These nutrient limiting conditions resulted in altered expression of several metabolism-associated genes and an increase of the unfolded protein response (UPR), witnessing a metabolic stress response (Additional file 1: Figure S1a-d) [28–30]. Of note, these conditions did not affect the viability of these two tumor cell lines (Additional file 1: Figure S1e-f). Strikingly, MHC-I surface display was strongly repressed in OGD cultured TC1 tumor cells (Fig. 1a-d). This effect was more pronounced in both tumor lines when the cells were stimulated with IFNy, a strong inducer of MHC-I antigen processing. Importantly, cells cultured under OD or GD conditions only did not repress MHC-I expression, indicating that the combination of both nutrients created a unique metabolic signature in tumor cells leading to repression of MHC-I. IFNy treatment did not affect this metabolic signature in the tumor cells as measured by the induction of metabolism-associated gene expression (Additional file 1: Figure S2a-b). The OGD-mediated resistance to IFNy was reversible as tumor cells transferred to normal nutrient-rich culture conditions restored the responsiveness to IFNy within 24 h (Fig. 1e and h).

To determine whether the decrease of MHC-I molecules was specific for IFNy signaling and not merely reflect a general reduction of surface proteins, we measured the expression levels of the cell surface glycoprotein CD44, and Tyrosinase-related protein-1 (TRP1). The expression of these two molecules was not downregulated, but rather somewhat upregulated under low-glucose conditions (Additional file 1: Figure S3a-c), excluding the possibility that OGD conditions led to a general reduction of cell surface protein expression.

To assess the functional impact of repressed MHC-I expression on tumor cells, we co-cultured the tumor cells with CD8 T cell clones specific for TC1 (CTL clone 9.5C3) [27] or B16F10 (CTL clone LP9) [26] tumor cells and measured T cell activation. The recognition by tumor-specific T cells was clearly reduced when OGD cultured tumor cells were used as targets, compared to normal or single-deprived culture conditions for either glucose or oxygen (Fig. 1f-g and h-i). In addition to the reduced frequency of T cells responding to the tumor targets, the degree of cytokine production was also strongly repressed. When the tumor targets were pre-incubated with IFNy to stimulate MHC-I levels [3] (Fig. 1b and d), the differences between the four culture conditions was even more pronounced, especially for the B16F10 tumor line (Fig. 1g and j). Taken together, these data indicated that tumor cells in a OGD environment repressed MHC-I surface expression, even after stimulation with IFNy, leading to strongly decreased immune recognition by CD8 T cells.

### Unresponsiveness to IFNy is mostly regulated via blockade of STAT1 phosphorylation

**Fig. 2:**
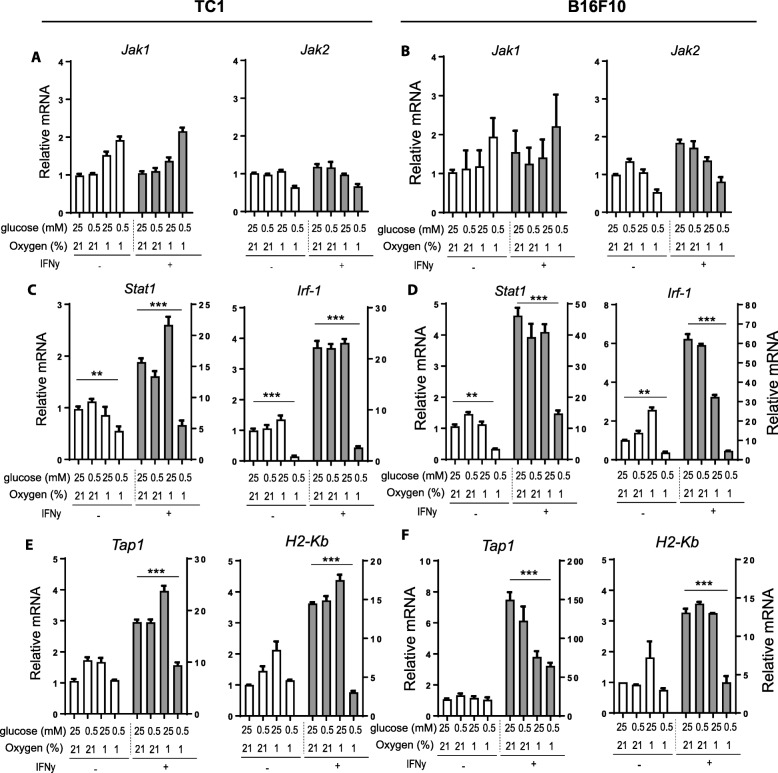
Gene transcription levels of IFNy-STAT1 relevant genes of tumor cells. Tumor cells were cultured under several nutrient limiting conditions for 24 h with or without IFNy. **a** and **b** *Jak1* and *Jak2* gene expression levels of TC1 (**a**) and B16F10 tumor cells (**b**) measured by qPCR. **c** and **d** *Stat1* and *Irf-1* gene expression levels of TC1 (**c**) and B16F10 tumor cells (**d**) measured by qPCR. **e** and **f** *Tap1* and *H2-K*^*b*^ (MHC-I) gene expression levels of TC1 (**e**) and B16F10 tumor cells (**f**) measured by qPCR. Relative mRNA expression is shown compared to normal culture conditions without IFNy stimulation and normalized to *UBC* housekeeping gene expression. Representative data is shown as mean + − SD (*n* = 3). Unpaired t test was used to calculate significance; n.s., not significant *, *p* < 0.05, **, *p* < 0.01, ***, *p* < 0.001

Our results indicate that the IFNy-STAT1 signal transduction pathway was impaired in OGD cultured tumor cells. This prompted us to determine the proficiency of the several components of IFNy-STAT1 signaling pathway of tumor cells under nutrient limiting conditions. In response to IFNy receptor (IFNyR) signaling, JAK1 and JAK2 are phosphorylated and subsequently STAT1 is phosphorylated at multiple residues, allowing dimerization, nuclear translocation and activation of interferon stimulated genes (ISGs) [31]. IFNyR surface expression on TC1 and B16F10 tumor cells and *Jak1* and *Jak2* expression was similar in all culture conditions (Additional file 1: Figure S4a-b and Fig. 2a-b, respectively). Strikingly, IFNy-mediated upregulation of *Stat1* expression levels were significantly inhibited in both TC1 and B16F10 tumor cells under OGD conditions (Fig. 2c-d). Moreover, the expression levels of *Interferon regulatory factor (Irf)-1*, and its downstream targets *Tap1* and *H2-K*^*b*^ (one of the MHC-I *genes)* were consequently also strongly inhibited in both tumor cell lines in OGD conditions (Fig. 2c-f).

**Fig. 3:**
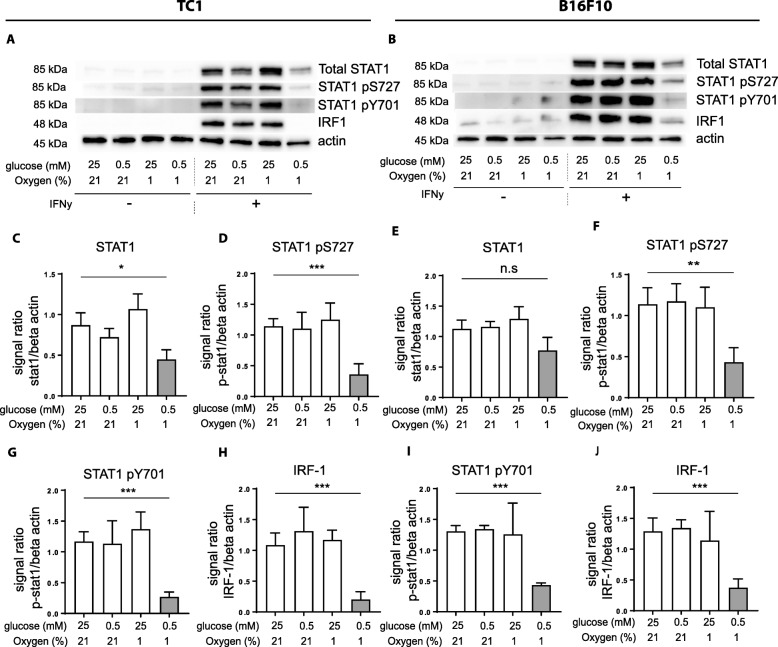
Impaired phosphorylation of STAT1 responsible for impaired MHC-I upregulation. Tumor cells were cultured for 24 h under several nutrient limiting conditions with or without IFNy. **a** and **b** Western-blots of TC1 (**a**) and B16F10 tumor cells (**b**). Representative data out of three experiments is shown. **c** and **e** Quantification of western-blot results of total STAT1 protein expression of TC1 (**c**) and B16F10 (**e**). **d**, **g** and **f**, **i** Quantification of western-blot results of phosphorylated STAT1 at Y701 and S727 of TC1 (**d**, **g**) and B16F10 (**f**, **i**). **h** and **j** Quantification of western-blot results of IRF-1 protein expression of TC1 (**h**) and B16F10 (**j**). Data was quantified by calculating signal ratio between protein of interest and beta-actin. Unpaired t test was used to calculate significance; n.s., not significant *, *p* < 0.05, **, *p* < 0.01, ***, *p* < 0.001

We then analyzed the IFNy-STAT1 signaling pathway at the protein level by Western-blot. IFNy stimulation greatly induced total STAT1 expression in normal, OG, and DG conditions in both tumor cell lines. When the tumor cells were cultured under OGD conditions, the upregulation of total STAT1 protein expression was inhibited, corroborating the qPCR results (Fig. 3a-b, c and e). More prominently, activation of STAT1 via tyrosine and serine phosphorylation was strongly repressed in OGD conditions upon IFNy stimulation (Fig. 3a-b, d and f, g and i). Consequently, protein expression of IRF-1 was virtually absent in tumor cells cultured under OGD conditions (Fig. 3a-b, h and j). Together these data show that OGD conditions affect the IFNy responsiveness of tumor cells, which is associated with strongly decreased STAT1 activation.

### Constitutive active STAT1 but not wild-type STAT1, rescues MHC-I expression in OGD culture conditions

**Fig. 4:**
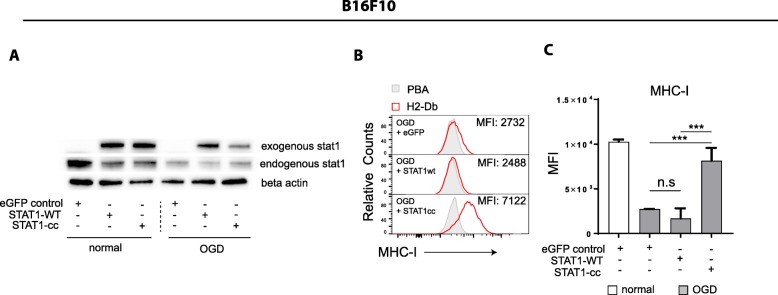
Transient expression of constitutive active STAT1 restores MHC-I expression on OGD stressed tumor cells. B16F10 tumor cells were transiently overexpressed with STAT1-WT or STAT1-cc using e-GFP as a control, prior to culturing the cells under normal or OGD culture conditions for 24 h with IFNy stimulation. **a** Western-blot results of endogenous STAT1 expression and exogenous STAT1 overexpression after transfection of STAT1-WT, STAT1-cc, or control. **b** Histogram illustrating MHC-I surface expression of B16F10 tumor cells cultured under normal or OGD conditions measured by flow cytometry. Representative data is shown as mean + − SD (*n* = 3). **c** Quantified data of B is shown as MFI. Unpaired t test was used to calculate significance; n.s., not significant *, *p* < 0.05, **, *p* < 0.01, ***, *p* < 0.001

STAT1 activation through phosphorylation is essential for downstream IFNyR signaling. We, thus, set out to determine the importance of STAT1 activation for the MHC-I presentation in OGD condition. Two different STAT1 constructs were introduced, one encoding the wild-type sequence of the gene (‘STAT1-WT’) and another with constitutive active STAT1 (‘STAT1-cc’) comprising point mutations in the src homology 2 (SH2)-homodimerization domain. This STAT1-cc mutant results in a IFNy hypersensitive variant of STAT1 [32]. Transient overexpression of STAT1-wt in B16F10 tumor cells that were cultured in OGD conditions led to strong increase in STAT1 protein levels (Fig. 4a), but failed to restore MHC-I expression (Fig. 4b-c). However, transient overexpression of the constitutive active STAT1-cc mutant restored MHC-I expression on B16F10 tumor cells cultured under OGD to levels, comparable to those of B16F10 cells cultured under normal conditions (Fig. 4b-c). These results implied that the OGD-mediated repression of MHC-I is mediated by inhibited phosphorylation of STAT1 affecting its downstream functions.

### OGD conditions activate AKT/PKB and chemical inhibitors for the upstream PI3K restored IFNy responsiveness of tumor cells

**Fig. 5:**
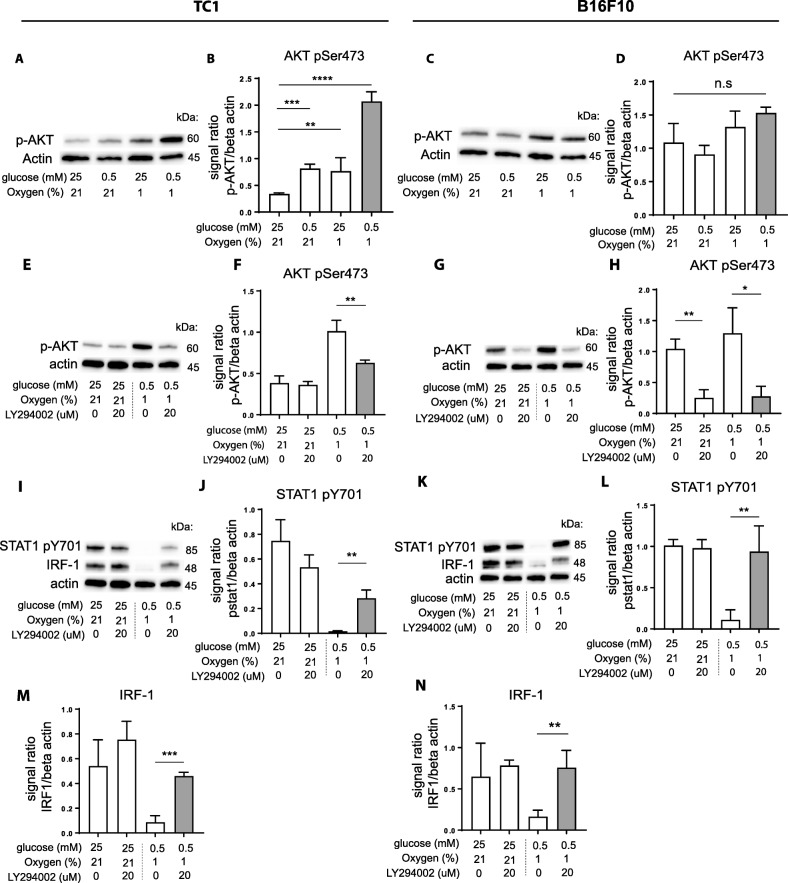
IFNy responsiveness of metabolically stressed tumor cells can be restored by PI3K inhibition. Tumor cells were cultured under normal, GD, OD, or OGD conditions together with IFNy for 24 h. **a** and **c** Western-blots of TC1 (**a**) and B16F10 (**c**) tumor cells. Representative data out of three experiments is shown. **b** and **d** Quantification of western-blot results of phospho-AKT expression in TC1 (**b**) and B16F10 (**d**) tumor cells. **e** and **g** TC1 (**e**) and B16F10 (**g**) tumor cells were cultured under normal or OGD conditions with or without LY294002 for 24 h. Representative western-blot results out of three experiments is shown. **f** and **h** Quantification of western-blot results of phospho-AKT expression in TC1 (**f**) and B16F10 (**h**) tumor cells. **i** and **k** Western-blots of TC1 (**i**) and B16F10 (**k**). Phospho-STAT1 and IRF-1 expression levels are shown. **j**, **l**, **m** and **n** Quantified data of (**i** and **k**). Data was quantified by calculating signal ratio between protein of interest and beta-actin. Unpaired t test was used to calculate significance; n.s., not significant *, *p* < 0.05, **, *p* < 0.01, ***, *p* < 0.001

We then investigated the mechanism by which OGD cultured tumor cells reduced their capacity to respond to IFNy, resulting in impaired STAT1 phosphorylation. Phosphoinositide 3-kinases (PI3Ks) play pivotal roles in the regulation of cellular metabolism [33, 34] and its downstream effector AKT/PKB mediates survival of tumor cells under hypoxia [35] and protects cells from death induced by glucose deprivation [36]. This involvement of PI3K in orchestrating metabolism prompted us to examine its role in IFNγ responsiveness under OGD conditions. Phosphorylation levels of the PI3K effector, AKT/PKB, in TC1 and B16F10 tumor cells were assessed by Western-blot and we indeed observed a strong upregulation of phospho-AKT (pAKT) under GD, OD and especially OGD conditions in the TC1 tumor cell line (Fig. 5a-b). A similar pattern was observed in the B16F10 tumor line, but less pronounced (Fig. 5c-d). These results showed that the PI3K pathway was activated under OGD conditions. To assess if upregulation of activated PI3K was responsible for the OGD-associated effects on IFNγ signaling we tested whether pharmacological inhibition of PI3K could restore the sensitivity of the tumor cells to respond to IFNγ. The common PI3K inhibitor LY294002 prevented phosphorylation of AKT induced by OGD conditions in both tumor cell lines, demonstrating functional inhibition of this pathway (Fig. 5e-h). Importantly, inhibition of PI3K signaling restored STAT1 Y701 phosphorylation and recovered protein expression of IRF-1 in TC1 cells (Fig. 5i-j, m) and B16F10 cells (Fig. 5k-l, n). In addition, pharmacological inhibition also restored total STAT1 protein levels in TC1 cells, but not in B16F10, most likely due to higher constitutive pAKT levels in these cells (Additional file 1: Figure S5a-d). We concluded that OGD culture conditions induced the activation of the PI3K/AKT pathway in tumor cells and thereby blocks IFNγ responsiveness.

### MHC-I surface display and CD8 T cell recognition are restored by inhibitors of PI3K

**Fig. 6:**
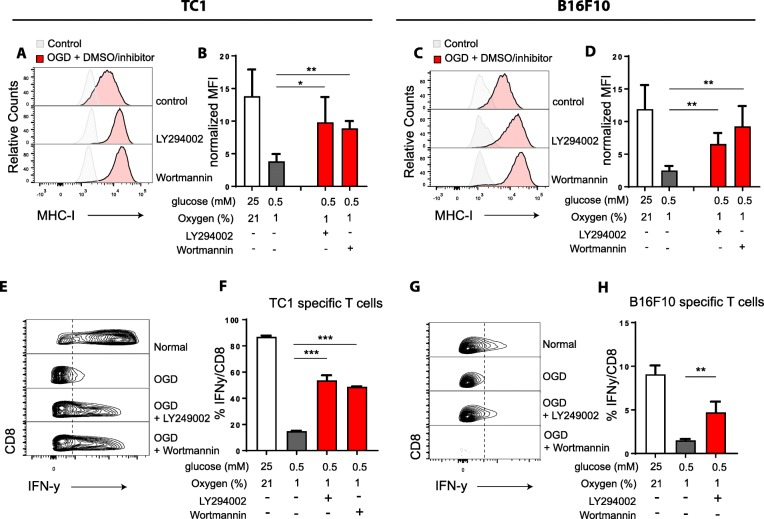
Improved tumor cell recognition by CD8 T cells after PI3K inhibition. Tumor cells were cultured under several nutrient limiting conditions stimulated with IFNy for 24 h with or without LY294002 or Wortmannin using DMSO as a control. **a** and **c** Histogram plot of MHC-I expression of TC1 (**a**) or B16F10 (**c**) tumor cells measured by flow cytometry. Representative data of three experiments is shown. **b** and **d** Quantified data of (**a** and **c**), shown as normalized MFI. **e** and **g** Density plots of IFNy production of TC1 (**e**) or B16F10 (**g**) specific T cells upon tumor cell recognition measured by flow cytometry. **f** and **h** Quantified data of (**e** and **g**). Representative data is shown as mean + − SD (*n* = 3). Unpaired t test was used to calculate significance; n.s., not significant *, *p* < 0.05, **, *p* < 0.01, ***, *p* < 0.001

Then, we investigated the impact of the PI3K inhibitors on MHC-I expression on tumor cells cultured under OGD conditions and compared this to tumor cells cultured under normal conditions (Fig. 6a-d). An almost complete rescue of cell surface MHC-I expression was observed on both cell lines. To corroborate these findings, we repeated this experiment with another PI3K inhibitor, Wortmannin and similar dose-response effects were observed (Additional file 1: Figure S5e-f), implying that the OGD-induced repression of MHC-I could be counteracted by inhibition of PI3K activity. Finally, we tested if the restored MHC-I levels led to full stimulation of tumor-specific CD8 T cells (Fig. 6e-h). Indeed, blockade of PI3K activity by LY294002 (L) or Wortmannin (W) significantly improved tumor cell recognition by both tumor-specific CD8 T cells.

Together, our data demonstrate that metabolic stress induces a PI3K-dependent response signature which inhibits the IFNy responsiveness of tumor cells. Consequently, tumor cell recognition by CD8 T cells is strongly impaired allowing tumor cells to escape immune control. These findings highlight the influence of nutrient availability in the tumor microenvironment on immunoregulation.

## Discussion

We showed that the combination of hypoxia and glucose deprivation of tumor cells, often found in microenvironments of solid tumors, results in lower tumor immunogenicity reflected by an impaired IFNy responsiveness, lowered MHC-I surface expression, and decreased recognition by tumor-specific CD8 T cells. This was caused by disruption of IFNy-mediated activation of the STAT1 signaling pathway, restricting the activation of its downstream target molecules, including IRF-1, TAP1, and MHC-I. As a result, tumors failed to efficiently present tumor antigens to CD8 T cells and consequently may escape from immunosurveillance or immunotherapy. Mechanistical studies suggested that metabolic stress-induced activation of the PI3K/AKT pathway was the underlying cause since the chemical inhibitors of the PI3K pathway, LY294002 and Wortmannin, relieved the inhibition of the IFNy-STAT1 signaling pathway, resulting in restored antigen presentation, and restored CD8 T cell recognition of the tumor cells. These data suggest an important interplay between STAT1 and PI3K, linking cancer metabolism with immunity. Our results furthermore indicate that immunotherapy-induced recruitment of tumor-specific CD8 T cells and local production of IFNy might not suffice for an effective tumor killing response.

Genetic alterations in metabolism pathways or insufficient oxygen levels often dictates cancer cells to utilize anaerobic glycolysis instead of OXPHOS. *Charni* et al. showed that forcing glycolytic cancer cells to utilize OXPHOS by DCA (dichloroacetate) treatment, results in upregulation of MHC-I through activation of the ERK5/MAPK pathway [37]. Similar findings were reported by *Catalan* et al., showing a correlation between the loss of ERK5 expression and reduced MHC-I expression in glycolytic leukemia cells and transformed fibroblasts [38]. MHC-I presentation was also altered upon activation of an UPR response. *Almeida* et al., showed that overexpression of UPR signaling transcription factors ATF6 (nATF6) and XBP-1 (sXBP-1) in hek293T cells results in reduced MHC-I presentation [39]. Importantly, only surface expression of MHC-I was inhibited, as total MHC-I expression was not altered. This can be explained by limited peptide availability for MHC-I binding as a result of repressed protein synthesis [40, 41]. Interestingly, in addition with our observations that metabolic stress reduces the responsiveness of tumor cells to IFNy and thereby leads to reduced MHC-I expression, these studies describe a mechanism that directly inhibit basal levels of MHC-I surface expression. Together, it shows that metabolic alternations of cancer cells and its impact on the TME can directly or indirectly modulate the MHC-I presentation through different pathways.

The interplay between the PI3K and STAT1 pathways is not extensively studied and only a limited number of studies reported on interactions and crosstalk of the two pathways. Nguyen et al. showed that phosphorylation of STAT1 at serine 727 after IFNy stimulation is required for activation of PI3K and AKT in T98G glioblastoma cells [42], whereas Mounayar et al. reported a study on PI3Kα-dependent activation of STAT1 phosphorylation at serine 727, resulting in regulation of human mesenchymal stem cell immune polarization [43]. However, we observed that metabolic stress-induced increase of PI3K activity results in impaired STAT1 phosphorylation. To the best of our knowledge, no reports implicate PI3K activation as a negative regulator for STAT1 signaling. These contradicting findings about the crosstalk between PI3K and STAT1 might be explained by the fact that we investigated the role of PI3K as a metabolic regulator upon nutrient deficiency, while others concluded that STAT1 serine-727 phosphorylation is affected by a kinase downstream of PI3K under nutrient proficient conditions. Together, these findings suggest a complex interplay between PI3K signaling and STAT1 expression.

Nutrient deprivation, such as low oxygen and glucose levels, activates AMPK [44], which suppresses biosynthetic processes in cells [45]. This regulator of metabolic stress responses dampens anabolic cell growth through inhibition of mTOR, the coordinator of metabolism, via diverse mechanisms among which the TSC2 complex. These pathways promote cell survival by preventing apoptosis in times of limited nutrient availability [46]. AMPK is also a key player in the homeostasis of cellular acetyl-CoA by inhibiting acetyl-CoA carboxylase (ACC) activity, responsible for the conversion of acetyl-CoA to malonyl-CoA [47]. Acetyl-CoA is a key metabolite that links metabolism with cell signaling and transcription [48]. In addition, acetyl-CoA is the universal donor for acetylation reactions [49], and cellular availability of this metabolite can affect histone- and protein-acetylation in both nucleus and cytoplasm [47, 50]. Interestingly, Krämer et al. revealed a link between acetylation and STAT1 signaling in that it counteracts IFNy induced STAT1 phosphorylation [51]. Although beyond the scope of this study, we speculate that AMPK activation may alter STAT1 protein acetylation as a result of cellular acetyl-CoA accumulation and, consequently, reduces the IFNy responsiveness through inhibition of STAT1 phosphorylation. However, the exact mechanism and the involvement of PI3K activity in this pathway remain elusive and is subject of further research.

The failure of cancer cells to respond to IFNy caused by acquired mutations in the IFNy-STAT1 signaling pathway is an important predictor for cancer progression and patient survival [5, 52–54]. Whole exome sequencing of refractory melanoma tumor lesions of patients initially responding to anti–programmed death 1 (PD-1) therapy revealed loss-of-function mutations in the IFNyR-associated genes Janus kinase 1 (JAK1) and Janus kinase 2 (JAK2). This type of acquired resistance led to failure to respond to IFNy and lack of HLA class I antigen presentation. In parallel, evaluation of primary resistance to anti-CTLA therapy in a cohort of 16 melanoma patients also revealed mutations in genes associated with IFNy signaling pathway in 12 patients, confirming that defects in the IFNy-STAT1 pathway provide resistance to immunotherapy [4]. Moreover, recent CRISPR/CAS9 screening studies implicated other genes involved in regulating IFNy sensitivity as a cancer immunotherapy target, namely, tyrosine-protein phosphatase non-receptor type 2 (PTPN2) and apelin receptor (APLNR) [6, 7]. They showed in preclinical models that altering the expression of these genes resulted in altered IFNy responsiveness and reduced efficacy of anti-CTLA4 blockade therapy in vivo. Overall, these studies emphasize the importance of IFNy signal transduction for immunotherapeutic success in cancer therapy.

## Conclusions

In our study, we showed that soft-wired alterations in the IFNy-STAT1 pathway, which were induced by limited nutrient availability in the tumor microenvironment, reduced the IFNy responsiveness and consequent MHC-I antigen presentation of cancer cells. This highlights that acquired resistance to IFNy is not only caused by loss-of-function mutations but can also be acquired through common features in the tumor microenvironment, like oxygen- and glucose-deprivation. This cancer cell extrinsic determinant of nutrient limitation might therefore impact the efficacy of immunotherapy. This suggests that improving the influx of nutrients by normalization of the tumor vasculature [55] or reducing the glucose consumption by targeting cancer metabolism could potentially greatly improve immunotherapeutic success.

## Additional files

Additional file 1: **Figure S1.** (A, B) mRNA expression of genes associated with glycolysis, OXPHOS and UPRin TC1 (A) and B16F10 (B) tumor cells. (C, D) PCR fragments of *total Xbp1* and *spliced Xbp1* of TC1 (C) and B16F10 (D) tumor cells +/- IFNy for 24 h. (E, F) Images of TC1 (E) and B16F10 tumor cells (F) cultured for 24 h under normal, OD, GD, or OGD. Magnification 100x. Data is shown as mean +/−SD (*n* = 3). **Figure S2.** Tumor cells were cultured under normal, OD, GD, or OGD and stimulated with IFNy for 24 h. (A, B) mRNA expression of genes associated with glycolysis, OXPHOS and UPR response regulation in TC1 (A) and B16F10 (B) tumor cells. Representative data is shown as mean +/−SD (*n* = 3). **Figure S3.** Tumor cells were cultured under normal, OD, GD, or OGD with IFNy for 24 h. (A, B) CD44 surface expression on TC1 (A) and B16F10 (B) tumor cells. (C) TRP1 surface expression on B16F10 tumor cells. Data is shown as mean +/−SD. (*n* = 3). **Figure S4.** Tumor cells were cultured under normal, OD, GD, or OGD with IFNy for 24 h. (A, B) Expression and quantification of the IFNyR on TC1 (A) and B16F10 (B) tumor cells. (*n* = 3). **Figure S5.** (A, C) STAT1 protein expression in TC1 (A) and B16F10 (C) cultured under normal, OG, DG, and OGD with IFNy for 24 h. Representative data of three experiments is shown (B, D) Quantified data of A and C. (E, F) MHC-I expression on OGD cultured tumor cells treated with increasing concentrations of LY294002 or wortmannin in TC1 (E) or B16F10 (F) tumor cells (*n* = 3). Unpaired t test was used for all experiments to calculate significance; n.s., not significant *, *p* < 0.05, **, *p* < 0.01, ***, *p* < 0.001. (PDF 427 kb)

Additional file 2: **Table S1.** Overview of primer sequences used for qPCR analysis. (DOCX 14 kb)

## Data Availability

Experimental data is available upon request.

## Abbreviations

GD: Glucose deprivation
IFNy: Interferon-gamma
MFI: Mean fluorescence index
MHC-I: Major histocompatibility complex-I
OD: Oxygen deprivation
OGD: Oxygen and glucose deprivation
TME: Tumor microenvironment
UPR: Unfolded protein response
