# Supplementary material for: Metabolic stress in cancer cells induces immune escape through a PI3K-dependent blockade of IFNγ receptor signaling
Source: J Immunother Cancer. 2019 Jun 13;7:152. doi: 10.1186/s40425-019-0627-8 (PMC6567539; doi:10.1186/s40425-019-0627-8)

Supplemental figure 1:

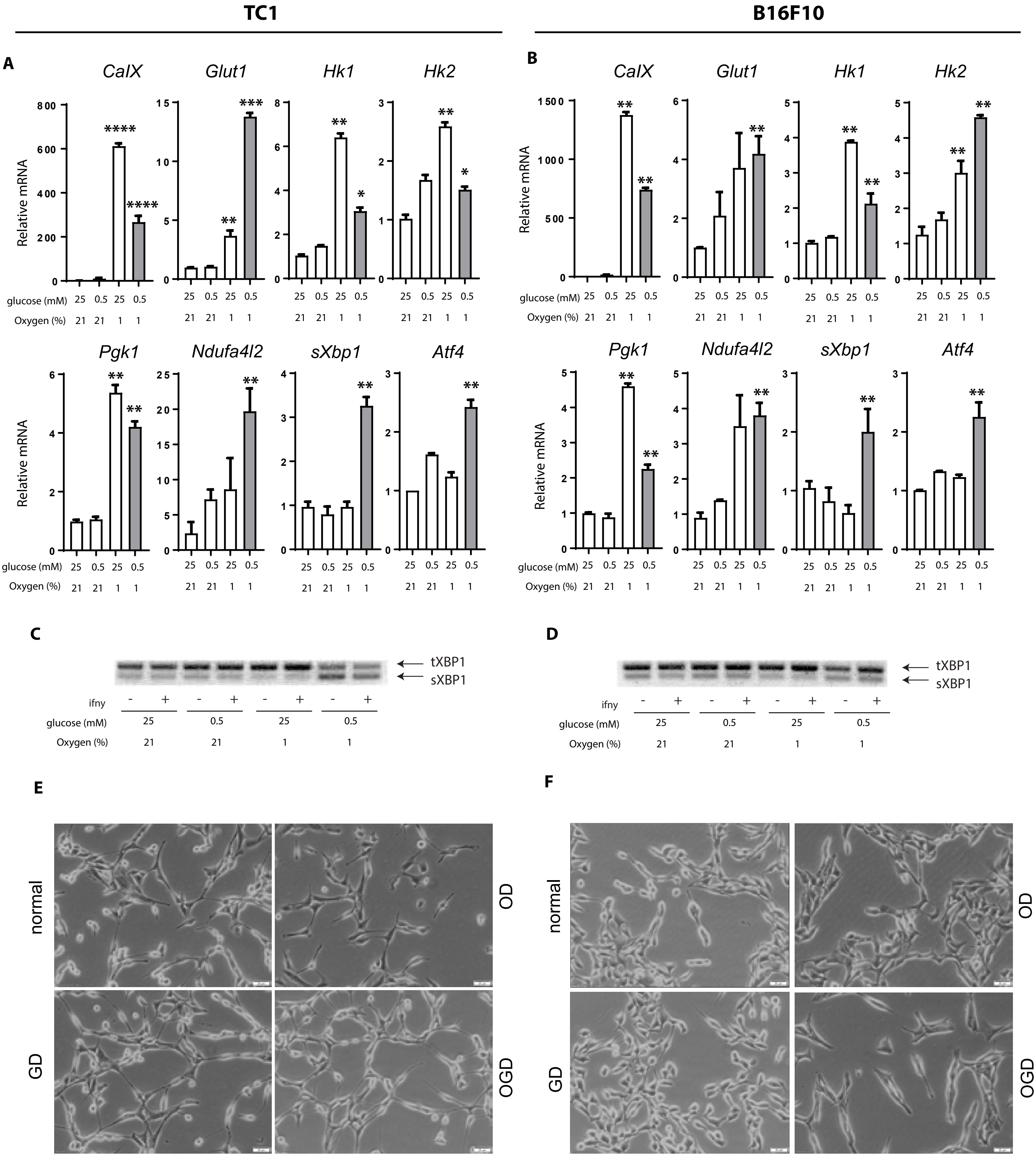

Supplemental figure 2:

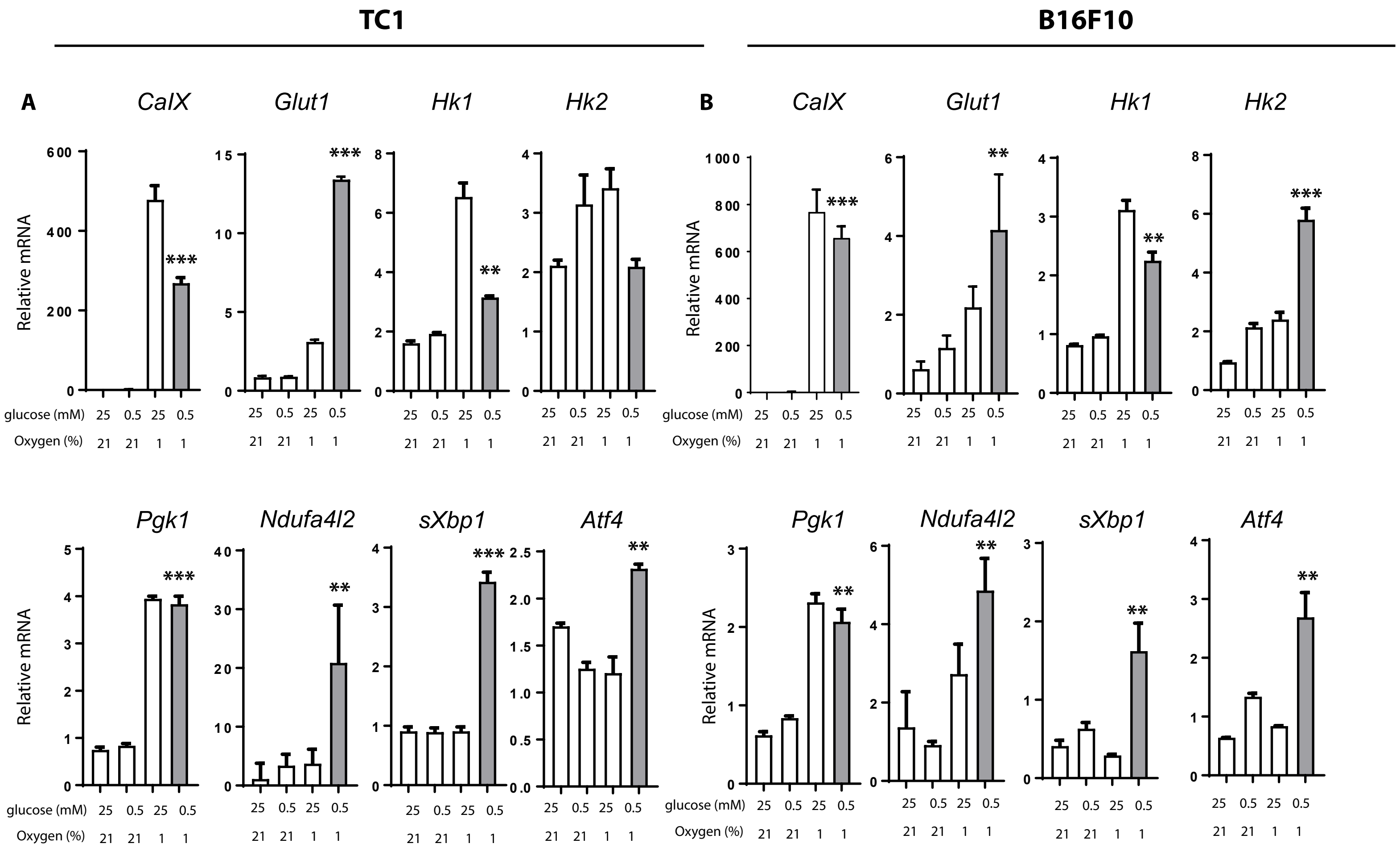

Supplemental figure 3:

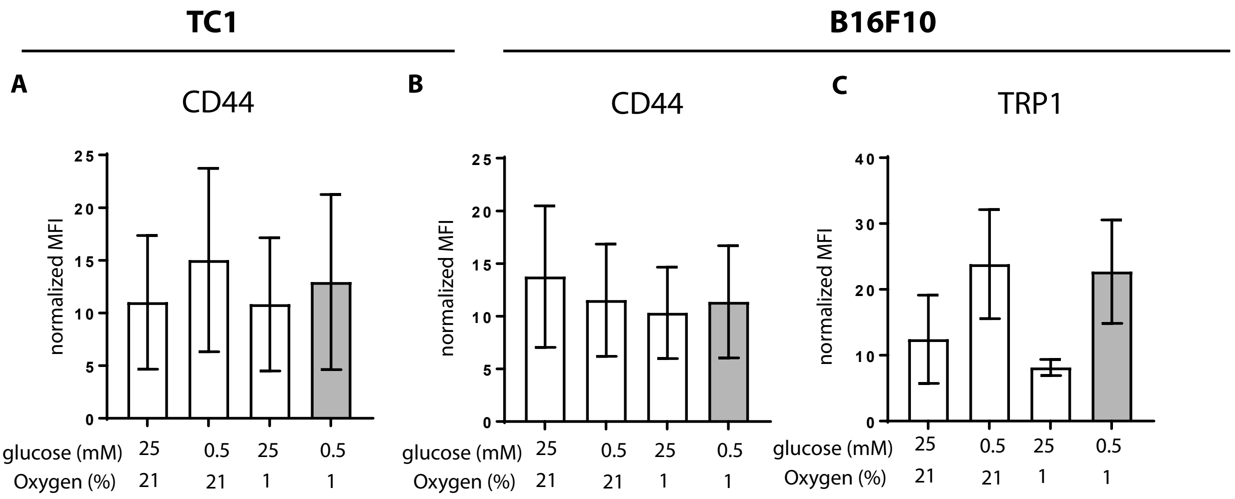

Supplemental figure 4:

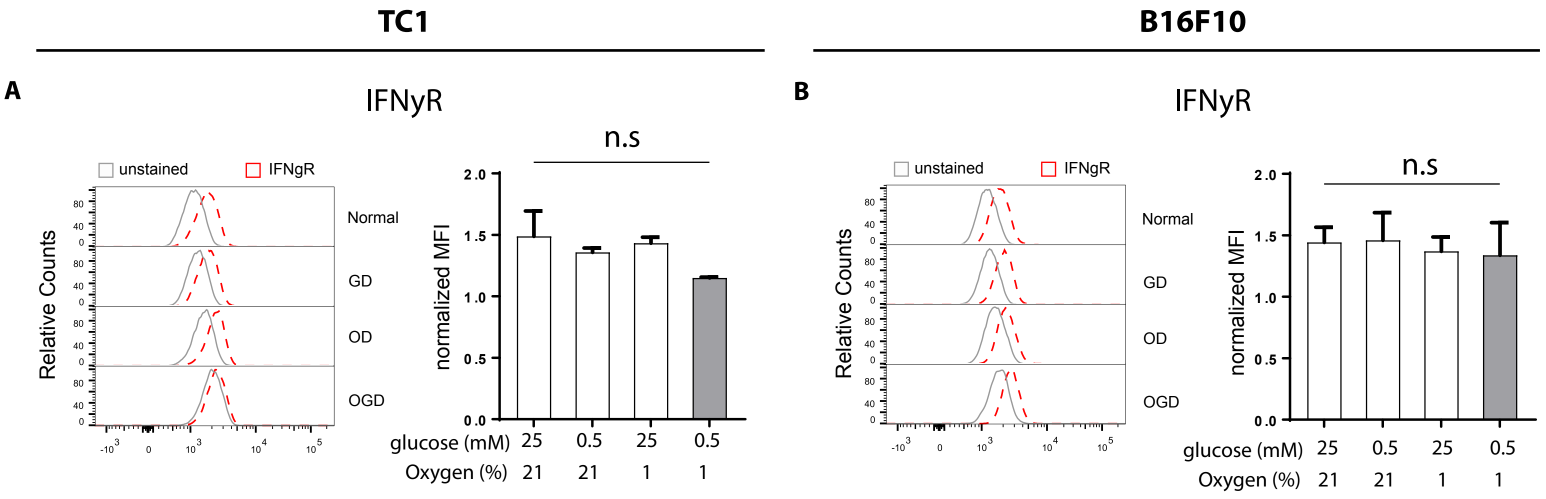

Supplemental figure 5:

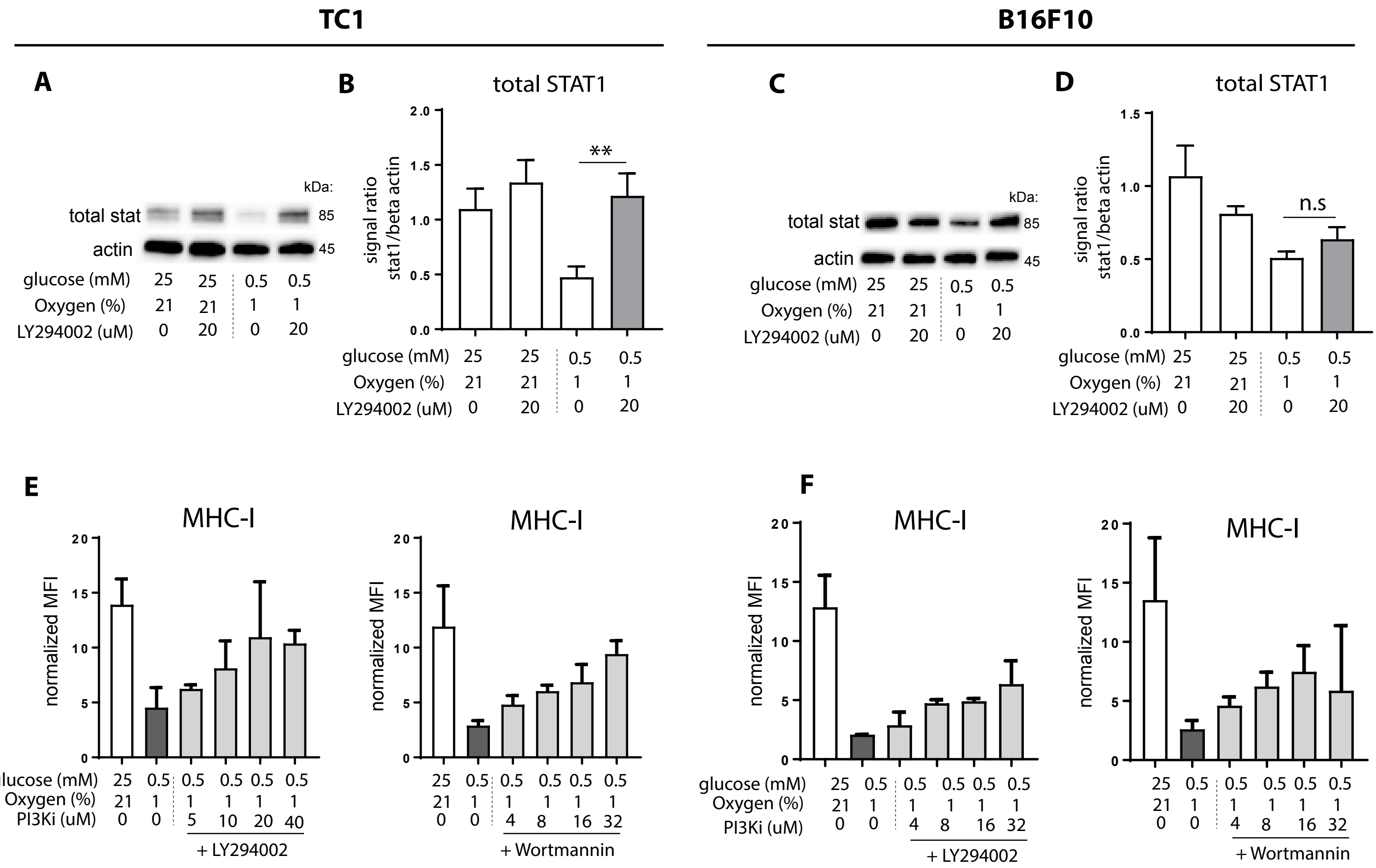

Supplement: Supplementary file 1 — Figure S1. (A, B) mRNA expression of genes associated with glycolysis, OXPHOS and UPRin TC1 (A) and B16F10 (B) tumor cells. (C, D) PCR fragments of total Xbp1 and spliced Xbp1 of TC1 (C) and B16F10 (D) tumor cells +/- IFNy for 24 h. (E, F) Images of TC1 (E) and B16F10 tumor cells (F) cultured for 24 h under normal, OD, GD, or OGD. Magnification 100x. Data is shown as mean +/−SD (n = 3). Figure S2. Tumor cells were cultured under normal, OD, GD, or OGD and stimulated with IFNy for 24 h. (A, B) mRNA expression of genes associated with glycolysis, OXPHOS and UPR response regulation in TC1 (A) and B16F10 (B) tumor cells. Representative data is shown as mean +/−SD (n = 3). Figure S3. Tumor cells were cultured under normal, OD, GD, or OGD with IFNy for 24 h. (A, B) CD44 surface expression on TC1 (A) and B16F10 (B) tumor cells. (C) TRP1 surface expression on B16F10 tumor cells. Data is shown as mean +/−SD. (n = 3). Figure S4. Tumor cells were cultured under normal, OD, GD, or OGD with IFNy for 24 h. (A, B) Expression and quantification of the IFNyR on TC1 (A) and B16F10 (B) tumor cells. (n = 3). Figure S5. (A, C) STAT1 protein expression in TC1 (A) and B16F10 (C) cultured under normal, OG, DG, and OGD with IFNy for 24 h. Representative data of three experiments is shown (B, D) Quantified data of A and C. (E, F) MHC-I expression on OGD cultured tumor cells treated with increasing concentrations of LY294002 or wortmannin in TC1 (E) or B16F10 (F) tumor cells (n = 3). Unpaired t test was used for all experiments to calculate significance; n.s., not significant *, p < 0.05, **, p < 0.01, ***, p < 0.001. (PDF 427 kb) [file 40425_2019_627_MOESM1_ESM.pdf]
