# Supplementary material for: Metabolic stress in cancer cells induces immune escape through a PI3K-dependent blockade of IFNγ receptor signaling
Source: J Immunother Cancer. 2019 Jun 13;7:152. doi: 10.1186/s40425-019-0627-8 (PMC6567539; doi:10.1186/s40425-019-0627-8)
Supplement: Supplementary file 2 — Table S1. Overview of primer sequences used for qPCR analysis. (DOCX 14 kb) [file 40425_2019_627_MOESM2_ESM.docx]

**Table S1**. Primers used for qPCR.

| Primer name | Primer sequence forward (5’-3’) | Primer sequence reverse (3’-5’) |
| --- | --- | --- |
| Ubc | GCCCAGTGTTACCACCAAGA | CCCATCACACCCAAGAACA |
| Stat1 | CCCCATGGAAATCAGACAGT | GAGAAAAGCGGCTGTACTGG |
| Tap1 | ATGCCTTCGCTATCAGTTA | GAGAATCAAGAGAACCAGAAC |
| H2k^b^ | GGAAGGAGCAGTATTACAC | CAAGGACAACCAGAACAG |
| H2d^b^ | ATCTCTGTCGGCTATGTG | TTGGCTTTCTGTGTTTCC |
| IFNyR | TTGAACCCTGTCGTATGCTG | TCAGAAATGTTGGTGCAGGA |
| CaIX | CACAGTCATTGGAGCTATGGAG | GCTGGAGCTCATAACCCAGA |
| Glut1 | GCAGTTCGGCTATAACACTGG | AACAAAGAGGCCGACAGAGA |
| Hk1 | AGATGCTGCCAACCTTTGTC | ACGATGTTCTCTGGGGTGTC |
| Hk2 | GAAGATGCTGCCCACCTTT | TTCTCCATCTCCACCCTCTG |
| Pgk1 | TAGAGCCAGTTGCTGCTGAA | GCATCTTTTCCCTTCCCTTC |
| Irf1 | ACCCTGGCTAGAGATGCAGA | AGGCATCCTTGTTGATGTCC |
| Ndufa4l2 | TGATTGGCTTCATCTGCTTG | ACTGGTCATTGGGACTCAGG |
| Atf4 | GCCGGTTTAAGTTGTGTGCT | CTGGATTCGAGGAATGTGCT |
| sXbp1 | GAGAACCAGGAGTTAAGAACACG | GAAGATGTTCTGGGGAGGTGAC |
| tXbp1 | GAATGGACACGCTGGATCCT | GCCACCAGCCTTACTCCACTC |
